# Psychological aspects of grazing in adolescents: Psychometric properties and measurement invariance of the Rep(eat)-Q in community and clinical samples

**DOI:** 10.1186/s40337-025-01344-5

**Published:** 2025-11-26

**Authors:** Sílvia Félix, Sofia Ramalho, João Marôco, Andreia Ribeiro, Nadine Afonso, Sónia Gonçalves, Eva Conceição

**Affiliations:** 1 Psychotherapy and Psychopathology Research Lab, Psychology Research Centre, School of Psychology, University of Minho, Campus de Gualtar, 4710-057 Braga, Portugal; 2 Faculty of Nutrition and Food Sciences of the University of Porto, Porto, Portugal; 3 Psychology for Development Research Center (CIPD), University of Lusíada, Porto, Portugal; 4 Intrepid Lab & CETRAD. ECEO., Lusófona University, Lisbon, Portugal; 5 Faculty of Psychology and Education Sciences, University of Porto, Porto, Portugal; 6 Center for Psychology at University of Porto, Porto, Portugal

**Keywords:** Grazing, Psychometric properties, Adolescence, Clinical sample, Community sample

## Abstract

**Background:**

Grazing is a disordered eating behavior associated with poor weight control, increased eating disorder psychopathology, and psychological difficulties in adults. Unfortunately, little is known about grazing in adolescence, which is aggravated by the lack of validated measures for this population. This study investigates the psychological aspects of grazing in adolescence and provides psychometric support for a brief self-report measure, the Rep(eat)-Q.

**Methods:**

A community sample of middle/high school students (n = 358, 55.6% females) and a clinical sample with overweight/obesity (n = 204, 59.8% females), completed a set of self-report questionnaires assessing eating and psychological variables including the Rep(eat)-Q. Weight and height data were also collected (Community: M_z-BMI_ = 0.39, SD = 0.98; Clinical: M_z-BMI_ = 2.39, SD = 0.74).

**Results:**

Confirmatory factor analysis revealed an adequate fit [(χ2(107) = 389.77; *p* < 0.001; CFI = 0.99; TLI = 0.99; NFI = 0.99; SRMR = 0.062; RMSEA = 0.098] for a second-order (grazing) model with two first-order factors (repetitive eating and compulsive grazing subscales) with good reliability (0.85 < α < 0.91). Metric and scalar invariance were confirmed, allowing comparisons between samples. Compared with clinical adolescents, community adolescents reported higher scores on the Rep(eat)-Q total (Community: M = 1.86, SD = 1.30; Clinical: M = 1.53, SD = 1.35; *t*(559) = − 2.81; *p* = 0.005) and repetitive eating subscale (Community: M = 2.04, SD = 1.44; Clinical: M = 1.52, SD = 1.35; *t*(560) = − 4.24; *p* ≤ 0.001). The Rep(eat)-Q total score and subscales scores were significantly positively correlated with eating disorder psychopathology and inversely correlated with intuitive eating, suggesting good convergent validity (0.11 < r < 0.63). Similarly, adolescents scoring higher on grazing also present more psychological distress and poor cognitive and emotional functioning (0.15 < r < 0.50). Psychological variables (i.e., depression, anxiety, and negative urgency) explained 21.8% of the variance in the grazing score, independent of sex, age, and BMI z-score [F(6, 490) = 22.87, *p* ≤ 0.001; R^2^ = 0.218].

**Conclusions:**

The Rep(eat)-Q is a reliable self-reported measure for assessing grazing in adolescents. These findings provide further support for the conceptualization of grazing in the spectrum of disordered eating and psychopathology in adolescents.

## Abstract (plain language summary)

This study explores grazing, a disordered eating behavior linked to poor weight control and psychological difficulties, in adolescents. It validates the Rep(eat)-Q, a brief self-report tool, to assess grazing in this age group. Researchers examined data from middle/high school students (n = 358) and adolescents with overweight/obesity (n = 204), analyzing eating behaviors, psychological variables, and BMI. Results confirmed that the Rep(eat)-Q reliably measures grazing, with two key subscales: repetitive eating and compulsive grazing. Community adolescents reported higher grazing scores than their clinical counterparts. Higher grazing scores correlated with disordered eating, psychological distress, and poor emotional functioning, while negatively correlating with intuitive eating. Psychological factors like depression, anxiety, and impulsivity explained 21.8% of the variance in grazing, independent of age, sex, and BMI. The study highlights the Rep(eat)-Q as a valid tool for identifying grazing in adolescents, supporting its role as part of disordered eating and psychopathology. This measure can aid early identification and intervention for adolescents struggling with grazing behaviors.

## Background

Disordered eating behaviors are common in adolescents and significantly affect weight control and psychopathology [21, 46]. Among adolescents with overweight or obesity, compared to those with normal-weight, disordered eating behaviors are more prevalent and appear to be associated with poor weight loss treatment outcomes, loss of control over eating, and a greater risk for the onset of an eating disorder later in adolescence or early adulthood [17, 22, 46].

Grazing is a disordered eating behavior that has received much attention over the past decade because of its association with eating disorder psychopathology, psychological distress, increased body mass index (BMI), and poor weight loss or weight management [2, 8, 23, 24, 31, 36]. Grazing is described as the ingestion of small/modest amounts of food in a repetitive and unplanned manner throughout a period of time, not in response to hunger/satiety sensations [7–9]. Two subtypes of grazing have been proposed: compulsive grazing, described as the inability to resist going back to eat, and non-compulsive grazing, characterized by eating in a distracted/mindless manner “on the spur of the moment”. Among these, compulsive grazing is considered more severe within the spectrum of eating disorder psychopathology because of its stronger association with the experience of loss of control eating [18].

Grazing has been investigated particularly among patients undergoing bariatric surgery or in adults from the community [7], but there is little research in the younger population. To the best of our knowledge, only one study has assessed grazing in a sample of adolescents and reported a negative association with intuitive eating [35]. Considering the increasing evidence for an association between grazing, weight loss in patients under treatment for obesity, and disordered eating psychopathology in adults, investigating whether these associations hold in the high-risk period of adolescence is timely in the field. Unfortunately, the lack of a validated measure to assess grazing in adolescents hinders the examination of grazing eating patterns in this population and its longitudinal association with the onset of obesity and eating disorders in this population.

A simple and short self-report measure was developed based on the definition proposed by Conceição and colleagues [7], the Repetitive Eating Questionnaire [Rep(eat)-Q] [8]. This measure assesses the repetitive nature of grazing as well as the compulsive nature of the behavior. Various studies have provided evidence for robust psychometric properties of the measure in community and clinical samples of adults, supporting significant correlations with disordered eating and psychological variables. Specifically, the Rep(eat)-Q has been validated in different languages, including European Portuguese [8], Brazilian Portuguese [40], Norsk [36], Turkish [1], Italian [38] and Chinese [25]. Unfortunately, no study has provided psychometric evidence for adolescents.

The aims of the present work are as follows: (1) To analyze the psychometric properties of the Rep(eat)-Q self-report measure for grazing in two samples of adolescents (i.e., a community and a clinical sample of adolescents undergoing hospital treatment for overweight/obesity); (2) To test measurement invariance and compare grazing between community and clinical samples; (3) To investigate associations between grazing and other eating-related psychological variables; and (4) To investigate if the psychological-related aspects of grazing behavior are dependent on sociodemographic, and antrophometric variables.

## Method

### Participants

This study included participants from two adolescent samples: a community sample and a clinical sample. The community sample included adolescents attending middle (7th, 8th, 9th grades) or high (10th, 11th, 12th grades) schools in three schools in northern Portugal, with ages ranging between 12 and 19 years. The clinical sample included adolescents undergoing medical weight loss treatment in two central hospitals in northen Portugal. The inclusion criteria for the clinical sample were being between 12 and 19 years of age and having a BMI for age above the 85th percentile. The exclusion criteria for both community and clinical samples were as follows: (1) not understanding written and spoken Portuguese and (2) presenting active developmental disorders, intellectual disabilities and/or psychiatric conditions that may interfere with participation (e.g., severe and nonmedicated attention deficit hyperactivity disorder).

## Procedure

The present study included adolescents from two independent broader studies (one with community sample and the other with clinical sample). Each study applied distinct recruitment processes and assessment measures. Data collection took place between January and November 2022 for the community sample and from October 2015 to October 2017 for the clinical sample.

Adolescents from the community population were recruited from middle and high schools. They received a document with all the information regarding the study goals and procedures. Written informed consent from the adolescents and parents/legal guardians was obtained prior to data collection. The research team then went to the school on previously scheduled days and assisted participants in completing a set of self-report questionnaires via the Qualtrics platform (or in paper–pencil format when online access was not possible). The self-report battery of questionnaires used in schools assesses grazing [Rep(eat)-Q], eating psychopathology (EDE-Q-7), negative urgency (UPPS), difficulties in emotion regulation (DERS), self-criticism (FSCRS), executive functioning (TEXI), and depression and anxiety symptoms (DASS-21). Weight and height were also measured by the research team.

Eligible adolescents from the clinical population were invited to participate during their previously scheduled medical appointment at the hospital centers. Parents and adolescents signed the informed consent form upon acceptance of participation. Subsequently, anthropometric data were collected, and the adolescents completed the clinical-sociodemographic questionnaire. The self-report questionnaires were collected online (via the Qualtrics platform) and grazing [Rep(eat)-Q], disordered eating behaviors (ChEAT), intuitive eating (IES-2), negative urgency (UPPS), quality of life (Peds-QL), and depression, anxiety, and stress symptoms (DASS-21) were assessed.

## Measures

### Anthropometric data

A SECA model 899 flat scale (SECA Corp., Hamburg, Germany, 2008) was used to collect weights (in kg) for the community sample. A Tanita^®^ TBF-300 Body Composition Analyzer was used to collect weights (in kg) from the clinical sample. Height (in cm) was assessed by the portable stadiometer Seca^®^ model 206. BMI z-scores for age and sex were calculated according to World Health Organization (WHO) Anthroplus software (macro for SPSS).

### Clinical-sociodemographic questionnaire

This self-report questionnaire was developed by the authors for the present study and collected information about adolescent participants and their parents/caregivers. It assesses adolescents’ age, biological sex, year of education in attendance, mental health problems, and parents’ education, marital status, and employment status.

### Repetitive eating questionnaire (Rep(eat)-Q) [8]

The Rep(eat)-Q was originally developed and validated in Portuguese adults. It is a 12-item self-report questionnaire that assesses the presence of grazing eating behavior. The respondents rated grazing frequency in the last 4 weeks using a 7-point ordinal scale [0 (never)–6 (every day)]. This questionnaire generates a total score and two subscales computed by the mean of the scale items: repetitive eating (items 1–4, 9, and 10) and compulsive grazing (items 5–8, 11, and 12). Higher scores indicate more frequent grazing eating behavior.

For the adaptation of the Rep(eat)-Q for adolescent, the adult version was applied to a small group of Portuguese adolescents, and a think-aloud reflection was conducted to assess the understanding of the questions. Since there were no doubts regarding the items, the adult version was used.

### Eating disorder examination questionnaire 7-item form (EDE-Q-7) [20, 29]

This 7-item version assesses disordered eating behaviors and attitudes (congruent with eating disorder psychopathology) focused on the past 28 days. Items are scored on a 7-point scale varying from 0 (“No days” or “Not at all”) to 6 (“Every day” or “Markedly”). It generates three scales: restraint, shape/weight overvaluation, and body dissatisfaction; and a total score. In the sample of this study, McDonald's ω was 0.83 for restraint, 0.90 for shape/weight overvaluation, and 0.90 for body dissatisfaction.

### Children's eating attitudes test (ChEAT) [30, 39]

This 26-item scale, answered on a scale from “never” to “always”, evaluates disordered eating attitudes (congruent with eating disorder psychopathology) through four subscales: (1) Fear of getting fat, (2) Restrictive and purging behaviors, (3) Preoccupation with food, and (4) Social pressure to eat. Higher scores indicating more disordered eating attitudes. In this study, McDonald's ω was 0.76 for fear of getting fat, 0.72 for restrictive and purging behaviors, 0.68 for preoccupation with food, 0.50 for social pressure to eat, and 0.81 for the total scale.

### Intuitive eating scale-2 (IES-2) [35, 42]

This scale assesses three main aspects of intuitive eating through a 16-item revised scale for adolescents with overweight obesity. It was answered on a 5-point Likert scale from “strongly disagree” to “strongly agree”: (1) eating for physical rather than emotional reasons, (2) reliance on internal hunger and satiety cues to determine when and how much to eat, and (3) body–food choice congruence. Higher scores indicate higher levels of intuitive (positive) eating. McDonald's ω for this study samples were 0.78, 0.20, 0.82, and 0.71 for eating for physical rather than emotional reasons, reliance on internal hunger, satiety cues, body-food choice congruence, and total score, respectively.

### Depression anxiety and stress scales (DASS-21) [28, 34]

This 21-item scale evaluates depression, anxiety, and stress symptoms with a 4-point Likert scale from “Did not apply to me at all” to “Applied to me very much, or most of the time”. In the sample of this study, McDonald's ω was 0.90 for depression scale, 0.88 for anxiety scale, and 0.90 stress scale.

### Urgency, premeditation, perseverance, sensation seeking Scale (UPPS) (UPPS): negative urgency subscale [45]

This subscale has 12 items answered on a 4-point ordinal scale from “strongly agree” to “strongly disagree” and assesses the individual’s tendency to give in to strong impulses when under negative emotions. Higher scores indicate more negative urgency. McDonald's ω was 0.88 for this study sample.

### Difficulties in emotion regulation scale (DERS [10, 19])

This 36-item questionnaire assesses the following emotion regulation dimensions: emotional acceptance, goal-directed behaviors when distressed, impulsivity control, emotional awareness, emotion regulation strategies, and emotional clarity. All items are answered on a 5-point ordinal scale, ranging from “almost never” to “almost always”. Higher scores suggest greater problems with emotion regulation. In the sample of this study, McDonald's ω was 0.91 for acceptance, 0.85 for goal-directed behaviors, 0.88 for impulsivity, 0.83 awareness, 0.90 for strategies, 0.77 for clarity and 0.94 for the total scale.

### The forms of self-criticizing/attacking and self-reassuring scale (FSCRS) [6, 16]

This is a 22-item self-report questionnaire that assesses how participants typically react when things go wrong for them on a 5-point ordinal scale 0 (“not at all like me”) to 4 (“extremely like me”). It generates three factors: inadequate self, hated self, and reassured self, with higher score indicating more of the respective domain assessed. In the sample of this study, McDonald's ω was 0.91 for inadequate self, 0.85 for hated self, and 0.88 reassured self.

### Teenage executive functioning inventory (TEXI) [41]

It uses a 5-point Likert scale ranging from 1 (“definitely not true”) to 5 (“definitely true”) to assesses two domains of executive functioning, namely, working memory and inhibition. Higher scores indicating greater difficulties in executive functioning. McDonald's ω was 0.88 for working memory and 0.83 for inhibition in the present study sample.

### Pediatric quality of life inventory (PedsQL^TM^) [27]

This is a 21-item measure of health-related quality of life in children and adolescents answered on a 5-point Likert scale from “never” to “always”. It generates a total score and two subscales: Psychosocial Health and Physical Health. Higher scores indicate quality of life. In this study, McDonald's ω was 0.90 for psychosocial health, 0.85 for physical health, and 0.92 for total score.

### Statistical analysis

Jamovi 2.3.28 (2023) and SPSS version 28 were used to perform statistical analysis. A significance level of 5% was used (α = 0.05) for all statistical analyses.

Calculations of measures of central tendency and dispersion were conducted. An exploratory analysis was performed, and data normality was tested. Severe univariate normality violations were considered for absolute values of skewness |sk|> 3 and kurtosis |ku|> 7 [15, 32].

A confirmatory factor analysis (CFA) was executed to assess the dimensional structure of Rep(eat)-Q. The Diagonally Weighted Least Squares estimation method was applied. Goodness-of-fit was evaluated using the NFI (Normed Fit Index), TLI (Tucker Lewis Index), CFI (Comparative Fit Index), RMSEA (Root Mean Square Error of Approximation), χ2 (chi-square statistic), χ2/df (chi-square statistic/degrees of freedom) and SRMR (Standardized Root Mean Square Residual). Criteria for a well-fitting model were defined as NFI, TLI, and CFI values > 0.95, and SRMR and RMSEA values < 0.08 [3, 5, 26, 33].

The reliability of the scores was evaluated using the internal consistency estimators α ordinal, with estimates ≥ 0.80 indicating satisfactory internal consistency evidence. The average variance extracted (AVE) was also calculated and values of AVE ≥ 0.50 were considered acceptable indicators of convergent evidence in terms of internal structure [32].

The measurement invariance among samples (i.e., community sample of adolescents vs. clinical sample of adolescents with overweight/obesity undergoing hospital treatment) was compared in several nested models: (1) configural invariance, (2) loadings (metric invariance), (3) thresholds (scalar invariance). Thresholds in detriment of intercepts were used to test scalar invariance because indicators were ordinal [44]. Invariance was confirmed if values of ΔCFI were ≤ 0.010 and/or the Δχ2 achieves non-significant *p*-values.

After confirming scalar invariance, Rep(eat)-Q second-order factor and Repetitive Eating and Compulsive Grazing subscales were compared across samples via T-tests for independent samples. The effect sizes were considered small, medium or large if Cohen’s *d* were ≥ 0.20, 0.50, or 0.80, respectively [14].

Pearson's correlation coefficients were performed to test associations between the Rep(eat)-Q (sub)scale(s) and other eating-related (convergent evidence) and psychological variables. Cohen’s effect size benchmarks were used to assess effect sizes classes for the correlations (i.e., small: ≥ 0.10; medium: ≥ 0.30; large: ≥ 0.50) [14]. Finally, to determine whether sociodemographic/anthropometric (i.e., sex, age, and BMI z-score) and psychological variables [i.e., negative urgency (UPPS), anxiety and depression symptoms (DASS-21)] were predictors of grazing [Rep(eat)-Q total score], a linear regression analyze was performed considering community and clinical samples together. The effect size was considered small, medium or large if r^2^ was ≥ 0.01, 0.09, or 0.25, respectively [14].

## Results

### Samples’ characterization

A total of 574 adolescents participated in the primary studies. Seven adolescents from the community sample and six from the clinical sample were excluded for not having responded to the Rep(eat)-Q, resulting in a final sample of 562 participants (n = 358 for the community sample, n = 204 for the clinical sample).

The community sample included 199 (55.6%) girls and 159 (44.4%) boys. The mean age was 15.60 (SD = 1.75) years; 275 (77.0%) adolescents were attending high school and 83 (23.0%) were attending middle school. The mean BMI was 22.17 kg/m^2^ (SD = 3.50), with a BMI for age z-score of 0.41 (SD = 0.98). According to the BMI for age z-score, most of the sample had normal weight (69.8%, n = 241) but 20.7% (n = 74) had overweight and 5% (n = 18) had obesity. About 49.2% of mothers and 30.4% of fathers completed high school or college education and the majority of the parents reported having a full-time job (87.4% for fathers and 83.8% for mothers).

The clinical sample included 122 (59.8%) girls and 82 (40.2%) boys. The mean age was 15.04 (SD = 1.63) years old; 51.5% (n = 105) were attending middle school and 28.5% (n = 99) were attending high school. The mean BMI was 31.12 kg/m^2^ (SD = 6.01), with a BMI for age z-score of 2.39 (SD = 0.74). Approximatly 27% of the participants from clinical sample were assessed at their first medical appointment for weight loss treatment, whereas the remaining participants were already engaged in ongoing follow-up, with heterogeneous durations. The mean follow-up duration was 787.36 days (SD = 1178.54). Twenty-five percent (n = 51) of the sample had overweight and 75% (n = 153) had obesity. Concerning parents’ information, 28.2% of mothers and 22.6% of fathers completed high school or college education and most parents reported having a full-time job (81% for fathers and 69.7% for mothers).

Table 1 displays the means and stantard deviations for all the self-reported measures applied to the community and clinical samples.

**Table 1:** Descriptive statistics of the self-reported measures assessed per sample

	Community sample		Clinical sample	
	M	SD	M	SD
Rep(eat)-Q total score	1.86	1.30	1.53	1.35
Rep(eat)-Q repetitive eating	2.04	1.44	1.52	1.35
Rep(eat)-Q compulsive grazing	1.67	1.43	1.54	1.54
EDE-Q-7 total score	1.94	1.70	–	–
EDE-Q-7 restraint	1.51	1.81	-	-
EDE-Q-7 SWO	2.12	2.12	–	–
EDE-Q-7 BD	2.41	2.11	–	–
ChEAT total score	–	–	16.08	9.10
ChEAT RPB	–	–	2.66	3.13
ChEAT FP	–	–	2.99	2.41
ChEAT FGF	–	–	9.77	5.78
ChEAT SPE	–	–	0.30	0.95
IES-2 total score	–	–	3.31	0.46
IES-2 EPER	–	–	3.45	0.90
IES-2 RHSC	–	–	3.20	0.40
IES-2 BFC	–	–	3.47	0.79
DASS-21 depression	6.25	5.21	5.52	5.43
DASS-21 anxiety	5.78	5.22	4.31	4.47
DASS-21 stress	–	–	6.29	5.24
UPPS—NU	2.43	0.57	2.51	0.60
DERS total score	92.01	24.26	–	–
DERS strategies	19.01	7.49	–	–
DERS non-acceptance	13.72	6.11	–	–
DERS awareness	16.97	5.22	–	–
DERS impulsivity	14.60	5.83	–	–
DERS goals	15.13	4.92	–	–
DERS clarity	12.58	4.45	–	–
FSCRS inadequate self	14.91	8.91	–	–
FSCRS hated self	4.08	4.61	–	–
FSCRS reassured self	19.06	7.48	–	–
TEXI working memory	2.66	0.78	–	–
TEXI inhibition	2.86	0.70	–	–
PedsQL total score	–	–	77.32	15.31
PedsQL psycho health	–	–	75.18	15.58
PedsQL physical health	–	–	81.33	16.57

n_Community sample_ = 357; n_Clinical sample_ = 204

*M* Mean; *SD* Standard deviation; *Rep(eat)-Q* Repetitive eating questionnaire; *EDE-Q-7* Eating disorder examination questionnaire 7-item form; *SWO* Shape/weight overvaluation; *BD* Body dissatisfaction; *TEXI* Teenage executive functioning inventory; *DERS* Difficulties in emotion regulation scale; UPPS-NU Urgency, premeditation, perseverance, sensation seeking scale—negative urgency subscale; *FSCRS* The forms of self-criticizing/attacking and self-reassuring scale; *DASS* Depression, anxiety, and stress scales; *ChEAT* Children’s eating attitudes test; *IES* Intuitive eating scale; *RPB* Restrictive and purgative behaviors; *FP* Food preoccupation; *FGF* Fear of getting fat; *SPE* Social pressure to eat; *EPER* Eating for physical rather than emotional reasons; *RHSC* Reliance on hunger an satiety cues; *BFC* Body-food congruence; *PedsQL* Pediatric quality of life inventory; *Psycho Health* Psychosocial health

### Evidence of the psychometric properties of the Rep(eat)-Q

Each of the 12 items of the Rep(eat)-Q presented responses in all categories of the ordinal scale (0–6). The items did not present severe violations of univariate normality (i.e., |sk| and |ku| values below 3 and 7, respectively). Item 12 (i.e., “Snacked on food when you were anxious, bored or lonely, or under other emotions”) had the highest mean value (M = 2.09; SD = 2.00) and item 8 (i.e., “Felt driven to eat”) had the lowest mean value (M = 1.34; SD = 1.68). Table 2 presents the items' distribution properties for both samples.

**Table 2:** Items’ distributional properties

Item	Combined samples							Community sample							Clinical sample						
	M	SD	Min	Mdn	Max	sk	ku	M	SD	Min	Mdn	Max	sk	ku	M	SD	Min	Mdn	Max	sk	ku
Item 1	1.95	1.99	0	1	6	0.82	− 0.60	2.27	2.03	0	2	6	0.56	− 0.99	1.41	1.80	0	1	6	1.45	1.09
Item 2	1.87	1.79	0	1	6	0.84	− 0.26	2.05	1.84	0	2	6	0.62	− 0.67	1.57	1.67	0	1	6	1.30	0.34
Item 3	1.90	1.87	0	1	6	0.80	− 0.48	2.02	1.81	0	2	6	0.58	− 0.74	1.67	1.96	0	1	6	1.17	0.15
Item 4	1.80	1.88	0	1	6	.89	− 0.36	2.01	1.85	0	2	6	0.64	− 0.75	1.44	1.88	0	1	6	1.44	0.93
Item 5	1.54	1.87	0	1	6	1.07	− 0.03	1.50	1.83	0	1	6	1.01	− 0.19	1.61	1.94	0	1	6	1.15	.14
Item 6	1.40	1.71	0	1	6	1.25	0.62	1.42	1.71	0	1	6	1.17	0.39	1.36	1.72	0	1	6	1.39	1.09
Item 7	1.69	1.78	0	1	6	1.01	− 0.00	1.75	1.76	0	1	6	0.89	− 0.29	1.60	1.82	0	1	6	1.23	0.55
Item 8	1.34	1.68	0	1	6	1.23	0.64	1.41	1.69	0	1	6	1.08	0.19	1.22	1.65	0	1	6	1.54	1.67
Item 9	1.76	1.85	0	1	6	0.89	− 0.31	1.85	1.90	0	1	6	0.76	− 0.56	1.59	1.77	0	1	6	1.14	0.32
Item 10	1.85	1.77	0	1	6	0.82	− 0.26	2.08	1.84	0	2	6	0.57	− 0.70	1.44	1.57	0	1	6	1.39	1.50
Item 11	1.70	2.06	0	1	6	1.00	− 0.40	1.58	2.00	0	1	6	1.01	− 0.38	1.90	2.13	0	1	6	0.96	− 0.50
Item 12	2.09	2.00	0	2	6	0.63	− 0.85	2.39	1.99	0	2	6	0.38	− 01.08	1.55	1.91	0	1	6	1.21	0.30

*N* 561; *M* Mean; *SD* Standard deviation; *Min* Minimum; *Mdn* Median; *Max* Maximum; *sk* Skewness; *k* Kurtosis

#### Validity evidence based on internal structure

Confirmatory factor analysis was used to test a second-order structure with two factors for the entire sample (considering clinical vs. community sample as the multigroup analysis factor). This second-order structure was chosen because the existence of a large amount of shared variance between the factors (i.e., repetitive eating and compulsive grazing) was previously shown to be better explained by the existence of a second-order factor (i.e. grazing) [40]. An adequate fit to the data was found (χ2(107) = 389.77, *p* < 0.001; χ^2^/df = 3.64; CFI = 0.99; TLI = 0.99; NFI = 0.99; SRMR = 0.062; RMSEA = 0.098; P(RMSEA ≤ 0.05) ≤ 0.001; 95% CI [0.087; 0.108]).

#### Evidence of construct validity and reliability

Both first-order factors showed acceptable evidence of internal consistency (compulsive grazing: α = 0.91; AVE = 0.69, and repetitive eating: α = 0.85; AVE = 0.58). The second-order factor also presented good internal consistency evidence (Rep(eat)-Q total: α = 0.86; AVE = 0.58).

### Measurement invariance and comparison between community and clinical samples

**Fig. 1:**
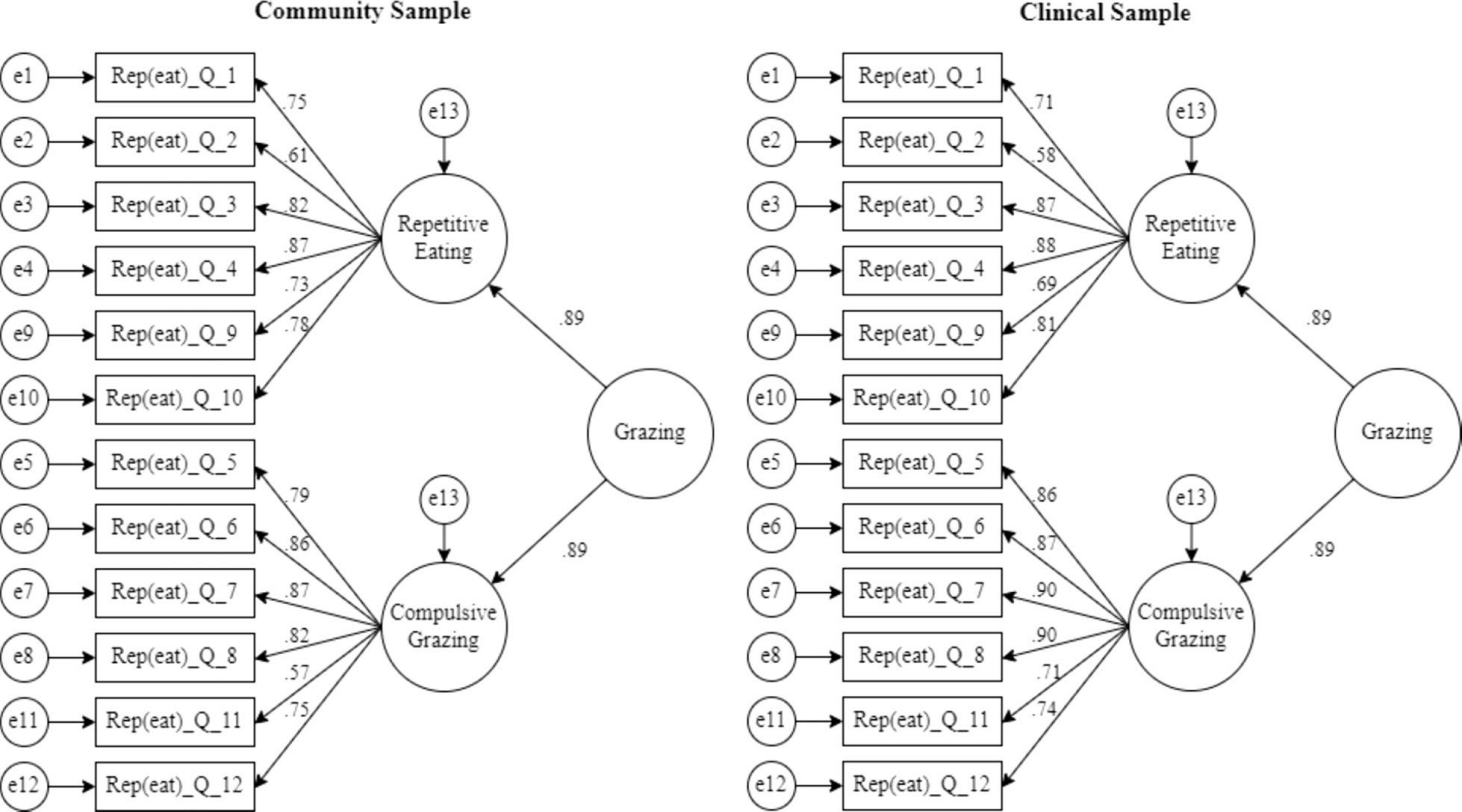
Factor loadings of the second-order model for the community and clinical sample

Figure 1 depicts the loadings for each sample. Table 3 presents the measurement invariance tests. The second-order model showed adequate fit indices, supporting configural invariance and indicating that the model fits both community and clinical samples. According to the criteria of Δχ2 and/or ΔCFI used to determine the fit of nested models, there is support for metric and scalar invariance. Metric invariance indicates that factor loadings and factor structure are identical across samples, and scalar invariance posits that thresholds are similar across groups, which allows the instrument scores to be compared among community and clinical samples.

**Table 3:** Tests of measurement invariance of Rep(eat)-Q for community and clinical samples

Model invariance	χ2	*df*	CFI	Δχ2	*p*(Δχ2)	ΔCFI
Configural	389.772	107	0.990	–	–	–
Metric	401.494	116	0.990	11.722	0.229	0.000
Scalar	605.129	173	0.985	203.635	≤ .001	0.005

*N* 561; *χ2* Chi-square statistic; *df* Degrees of freedom; *CFI* Comparative Fit Index

Community adolescents scored significantly higher than clinical adolescents in the Rep(eat)-Q total score [*t* (559) = − 2.81; *p* = 0.005; Cohen’s *d* = − 0.25] and in the Repetitive Eating Subscale [*t* (560) = − 4.24; *p* ≤ 0.001, Cohen’s *d* = − 0.37], but the effect sizes were small. No differences were found between the samples for Compulsive Eating subscale [*t*(560) = − 1.01; *p* = 0.312, Cohen’s *d* = − 0.09].

### Association with psychological/behavioral variables: convergent validity

Table 4 shows the correlations between the Rep(eat)-Q and the other variables under study for community and clinical samples.

**Table 4:** Pearson's correlations between the Rep(eat)-Q and sociodemographic variables, BMI z-score, and eating and psychological variables

	Rep(eat)-Q total score	Repetitive eating subscale	Compulsive grazing subscale
*Community sample*			
Age	0.121*	0.109*	0.106*
z-score BMI	0.078	− 0.052	0.196***
EDE-Q-7 total score	0.286***	0.107*	0.411***
EDE-Q-7 restraint	0.173***	0.001	0.313***
EDE-Q-7 SWO	0.332***	0.196***	0.409***
EDE-Q-7 BD	0.252***	0.110*	0.349***
TEXI working memory	0.379***	0.305***	0.383***
TEXI inhibition	0.443***	0.355***	0.450***
DERS total score	0.329***	0.226***	0.370***
DERS strategies	0.360***	0.258***	0.394***
DERS non-acceptance	0.299***	0.217***	0.324***
DERS awareness	− 0.071	− 0.089	− 0.040
DERS impulsivity	0.232***	0.164**	0.257***
DERS goals	0.345***	0.261***	0.367***
DERS clarity	0.148**	0.075	0.195***
UPPS—NU	0.428***	0.326***	0.453***
FSCRS inadequate self	0.370***	0.253***	0.420***
FSCRS hated self	0.346***	0.227***	0.402***
FSCRS reassured self	− 0.154***	− 0.098	− 0.181***
DASS-21 depression	0.378***	0.281***	0.406***
DASS-21 anxiety	0.369***	0.272***	0.399***
*Clinical sample*			
Age	0.061	0.095	0.013
z-score BMI	0.063	0.058	0.059
ChEAT total score	0.330***	0.199**	0.401***
ChEAT RPB	− 0.157*	− 0.200**	− 0.099
ChEAT FP	0.605***	0.485***	0.633***
ChEAT FGF	0.313***	0.184*	0.30.85***
ChEAT SPE	0.108	0.135	0.071
IES-2 total score	− 0.313***	− 0.292***	− 0.291***
IES-2 EPER	− 0.441***	− 0.390***	− 0.429***
IES-2 RHSC	0.041	0.043	0.034
IES-2 BFC	− 0.141*	− 0.181*	− 0.088
PedsQL total score	− 0.461***	− 0.350***	− 0.499***
PedsQL psycho health	− 0.447***	− 0.329***	− 0.494***
PedsQL physical health	− 0.386***	− 0.314**	− 0.400***
UPPS—NU	0.413***	0.366***	0.400***
DASS-21 depression	0.381***	0.265***	0.434***
DASS-21 anxiety	0.337***	0.227*	0.392***
DASS-21 stress	0.327***	0.217**	0.383***

n_Community Sample_ = 357; n_Clinical Sample_ = 204

Sex (1 = Female; 2 = Male); *BMI* Body mass index; *EDE-Q-7* Eating disorder examination questionnaire 7-item form; *SWO* Shape/weight overvaluation; *BD* Body dissatisfaction; *TEXI* Teenage executive functioning inventory; *DERS* Difficulties in emotion regulation scale; *UPPS-NU* Urgency, premeditation, perseverance, sensation seeking scale—negative urgency subscale; *FSCRS* The forms of self-criticizing/attacking and self-reassuring scale; *DASS* Depression, anxiety, and stress scales; *ChEAT* Children’s eating attitudes test; *IES* Intuitive eating scale; *RPB* Restrictive and purgative behaviors; *FP* Food preoccupation; *FGF* Fear of getting fat; *SPE* Social pressure to eat; *EPER* Eating for physical rather than emotional reasons; *RHSC* Reliance on hunger an satiety cues; *BFC* Body-food congruence; *PedsQL* Pediatric quality of life inventory; *Psycho Health* Psychosocial health

Correlation with medium or large effect size were highlighted in bold

^*^p < 0.05; **p < 0.005; ***p ≤ 0.001

Concerning associations with dimentions of eating behavior, as expected, the Rep(eat)-Q total score and its subscales mostly presented significant small to moderate correlations with eating disorder psychopathology in the community (EDE-Q-7) and clinical (ChEAT) samples and with intuitive eating (IES-2) in the clinical sample, showing evidence of good convergent validity.

There were also significant correlations with cognitive/behavioral measures for both community and clinical samples. In community samples, higher values of the Rep(eat)-Q total score and subscales, were associated (medium effect sizes) with greater difficulties in working memory, inhibition (TEXI), and negative urgency (UPPS). Similarly, the Rep(eat)-Q total score and compulsive grazing were positively correlated with self-criticism (hated self and inadequate self subscales of the FSCRS).

Emotion regulation was only assessed in the community sample. Higher values of the Rep(eat)-Q total score and its subscales were associated (small-to-medium effect sizes) with greater difficulties in DERS total score and its subscales of strategies, non-acceptance, goals, impulsivity and clarity (except for repetitive eating).

Regarding psychological distress, in both the community and clinical samples, higher values of the Rep(eat)-Q total score and its subscales were associated (with small-to-medium effect sizes) with depression, anxiety, and stress symptoms (DASS-21). There were also positive medium effect size correlations between Rep(eat)-Q total score and the health-related quality of life (PedsQL) scores for the clinical sample.

### Grazing in adolescents: the role of sociodemographic/anthropometric, and psychological variables

To investigate the role of sociodemographic, anthropometric and psychological variables in explaining grazing eating behavior the variables collected for the entire sample (i.e., combining community and clinical samples) were tested together as indicators of grazing (total sample size N = 574). Sex, age, and BMI z-score were entered in Step 1, followed by anxiety (DASS-21), depression (DASS-21), and negative urgency (UPPS) in Step 2. The results are displayed in Table 5.

**Table 5:** Linear regression analysis to test sociodemographic/anthropometric and psychological variables as predictors of grazing [Rep(eat)-Q total score)] for the complete sample (i.e., combining community and clinical samples)

	B	SE	β	*t*	95% CI	R^2^	F
*Setp 1*							
Sex	− 0.140	0.117	− 0.054	− 1.20	[− 0.141; 0.035]	0.013	2.22
Age	0.071	0.035	0.092	2.02*	[0.003 0.181]		
z-score BMI	− 0.025	0.045	− 0.025	− 0.055	[− 0.115; .065]		
*Step 2*							
Sex	0.188	0.108	0.071	1.73	[− 0.010; 0.154]	0.212	22.87***
Age	0.032	0.032	0.041	1.00	[− 0.040; 0.123]		
z-score BMI	− 0.063	0.040	− 0.029	− 1.56	[− 0.145; 0.017]		
DASS-21 Anxiety	0.038	0.016	0.146	2.44*	[0.029; 0.263]		
DASS-21 Depression	0.030	0.015	0.125	2.02*	[0.003; 0.246]		
UPPS—Negative Urgency	0.688	0.104	0.305	6.64***	[0.214; 0.395]		

*N* 504; Sex (1 = Female; 2 = Male); *BMI* Body mass index; *DASS* Depression, anxiety, and stress scales; *UPPS-NU* Urgency, premeditation, perseverance, sensation seeking scale—negative urgency subscale

^*^p < 0.05; ** *p* < 0.005; ***p ≤ 0.001

The first block, which included the sociodemographic and anthropometric variables sex, age, and BMI z-score, was not statistically significant [F(3, 493) = 2.22, *p* = 0.085, R^2^ = 0.013; small effect size]. When negative urgency, depression and anxiety symptoms were added to Block 2, the overall model was significant [F(6, 490) = 22.87, *p* ≤ 0.001)] and explained 21.8% of the variance in grazing (R^2^ = 0.218; medium effect size). This represents a statistically significant improvement in the explained variance [ΔR^2^ = 0.025; F(3, 490) = *p* ≤ 0.001]. Only the Block 2 variables were statisticaly significant indicators (i.e., depression, anxiety, and negative urgency). Interestingly, negative urgency was the strongest indicator, and for each unit increase in negative urgency symptoms, the grazing scores increased by 0.688 (β = 0.305). Age, sex and BMI z-score were not significant in the model.

## Discussion

This is the first study to investigate the psychometric properties of a measure to assess grazing eating behavior, the Rep(eat)-Q, in adolescents. Overall, the findings indicate that the Rep(eat)-Q presents adequate psychometric properties in both community and clinical samples of adolescents with overweight/obesity. Specifically, the instrument showed good validity evidence based on internal structure, construct validity, reliability, and its associations with other variables.

### Factor structure and scores for community and clinical samples

Our findings support a model with two first-order factors (repetitive eating and compulsive grazing), and a second-order latent factor of grazing as previously proposed by Teodoro and colleagues [40]. Both the first and second-order factors showed good reliability estimates and all the original items were retained. Furthermore, the analysis confirmed measurement invariance across community and clinical adolescents, allowing comparisons of the Rep(eat) scores across these samples.

Unexpectedly, the mean scores on the Rep(eat)-Q total (Community: M = 1.86, SD = 1.30; Clinical: M = 1.53, SD = 1.35) and repetitive eating subscale (Community: M = 2.04, SD = 1.44; Clinical: M = 1.52, SD = 1.35) were higher in the community sample than in the clinical sample. Adolescents in the clinical sample were undergoing hospital treatment for overweight/obesity, which primarily involved following a prescribed nutritional plan. This might have helped to reduce grazing by contributing to an increased awareness of undesirable behaviors. However, there were no significant differences in the scores of the compulsive grazing subscale between these two samples (Community: M = 1.67, SD = 1.43; Clinical: M = 1.54, SD = 1.54). This outcome is likely due to compulsive grazing involving an increased degree of loss of control over eating, which makes this behavior more severe and more resistant to change [18], even for individuals undergoing medical or nutritional interventions.

### Associations between grazing and psychological factors

The Rep(eat)-Q total score and its subscales were significantly associated with the other eating-related measures included in the study. Specifically, positive correlations were found with eating disorder psychopathology in both community and clinical samples, and negative correlations were found with intuitive eating in the clinical sample. These findings support convergent validity, further substantiating grazing as a construct of disordered eating behavior. Significant correlations were also found with other psychological measures in both the community and the clinical samples. Consistent with previous studies [8, 23, 24, 37], this study revelead that grazing was associated with higher levels of depression, anxiety, and stress symptoms,negative urgency; and difficulties in emotion regulation, as well as with lower quality of life in adolescents.

Notably, our findings introduce further novelty to the concept of grazing by exploring its correlation with new variables such as executive functions and self-criticism (assessed in the community sample). On the one hand, the existence of strong positive correlations between grazing and executive functions (i.e., working memory and inhibition) adds to the existing evidence that top-down mechanisms play a role in the regulation of eating behaviors [11]. On the other hand, the strong correlations between Rep(eat)-Q total and compulsive grazing with self-criticism (i.e., inadequate-self and hated-self subscales) align with previous evidence on binge eating (i.e., an eating behavior highly characterized by loss of control eating) [12, 13]. This evidence strengthens the understanding that disordered eating behaviors are related to psychological, cognitive and emotional difficulties.

Overall, the compulsive grazing subscale exhibited stronger correlations with other eating behaviors and psychological variables than did the repetitive eating subscale in both samples. The model proposed by Conceição and colleagues (2014) places various problematic eating behaviors on a continuum scale of loss of control over eating. According to this model, compulsive grazing is conceptualized as being higher in the spectrum of loss of control eating and eating-related psychopathology than grazing without loss of control, which has been supported by other subsequent studies [9, 23]. Thus, our findings bring further support for this conceptualization of grazing in a spectrum of disordered eating and extend the current literature to the developmental age of adolescents.

### Importance of sociodemographic and antrophometric variables

Finally, the linear regression analysis emphasized the importance of psychological variables over sociodemographic and anthropometric characteristics in understanding grazing in adolescents. Specifically, depression and anxiety symptoms, as well as negative urgency, were significantly associated with grazing, whereas sex, age, and BMI z-score were not. Among them, negative urgency was the strongest indicator, suggesting that adolescents scoring higher in grazing have a greater tendency to act impulsively in response to negative emotions. This finding is consistent with prior studies highlighting the central role of impulsive traits and loss of control in disordered eating behaviors [4, 9, 43], and underscores the importance of addressing emotional aspects and impulsive traits in interventions aimed at managing grazing. This may be particularly relevant in adolescence because this age group is particularly vulnerable to developing disordered eating behaviors and weight problems [17, 22, 46].

### Limitations

One limitation of our study is that, although the overall model fit was acceptable, the RMSEA value was close to the upper threshold, which warrants caution in interpreting the results. The temporal stability of the Rep(eat)-Q was not assessed in this study, despite our expectation of good test–retest reliability based on the original study. Additionally, the use of self-reported measures did not address social desirability bias, potentially biasing (by underreporting) the responses of patients from the clinical sample. The use of different measures across the two samples limits direct comparability. Also, the clinical sample was assessed at different stages of intervention, which may have influenced their psychological and behavioral profiles. Finally, approximately 23% of community sample participants fulfilled the questionnaires in paper–pencil format due to difficulties with online access, which compromised the uniformity of the procedures, but increased the validity across different response systems.

## Conclusion

The present study supports the use of the Rep(eat)-Q as a self-report measure with adequate psychometric properties to assess grazing in both community and clinical samples of adolescents. Our findings support a model with two first-order factors (repetitive eating and compulsive grazing), and a second-order latent factor of grazing. The scales possessed validity evidence based on internal structure, good reliability, and construct validity. This work also provides further evidence that grazing is a problematic eating behavior with clinical relevance among adolescents because of its association with other eating-related and psychological variables, which are independent of age, gender and BMI for this age group. This is also the first study to show that grazing is positively correlated with poor executive functioning and increased self-criticism, strengthening the role of cognitive and emotional variables in the understanding of disordered eating behaviors. The use of the Rep(eat)-Q in this population can help to screen grazing patterns and explore underlying mechanisms related to the development and maintenance of eating disorders and overweight/obesity. Future research should employ longitudinal designs to explore the prospective validity of grazing as a risk factor for the development of other disordered eating behaviors, psychological difficulties and/or weight fluctuations over time.

## Data Availability

The data that support the findings of this study are available from the corresponding author upon reasonable request.
